# The PID Life Index: an interactive tool to measure the status of the PID healthcare environment in any given country

**DOI:** 10.1186/s13023-021-02161-0

**Published:** 2022-01-08

**Authors:** Leire Solís, Julia Nordin, Johan Prevot, Nizar Mahlaoui, Silvia Sánchez-Ramón, Adli Ali, Elodie Cassignol, John W. Seymour, Martine Pergent

**Affiliations:** 1 The International Patient Organisation for Primary Immunodeficiencies, Downderry, UK; 2 Pediatric Immunology-Hematology and Rheumatology Unit, Necker Children’s University Hospital, Assistance Publique-Hôpitaux de Paris (AP-HP), Paris, France; 3 French National Reference Center for Primary Immune Deficiencies (CEREDIH), Necker Children’s University Hospital, Assistance Publique-Hôpitaux de Paris (AP-HP), Paris, France; 4 Department of Clinical Immunology, Instituto de Medicina del Laboratorio and Instituto de Investigación Clínico San Carlos, Hospital Clínico San Carlos, Madrid, Spain; 5 Department of Immunology, ENT Ophthalmology, Complutense University School of Medicine, Madrid, Spain; 6 Clinical Immunology Unit, Department of Paediatrics, Faculty of Medicine, Universiti Kebangsaan Malaysia Medical Centre, Kuala Lumpur, Malaysia; 7 Institute of IR4.0, Universiti Kebangsaan Malaysia, Bangi, Malaysia; 8 Quadrature du Cercle, Dataviz Agency, Paris, France; 9 Department of Counseling and Student Personnel, Minnesota State University, Mankato, MN USA

**Keywords:** Primary immunodeficiency, PID principles of care, PID life index, Data aggregation, Web-based resources, Health care policy, Rare diseases

## Abstract

**Background:**

The “Primary Immunodeficiencies (PIDs) principles of care” were published in 2014 as the gold standard for care of patients with PIDs, setting a common goal for stakeholders to ensure that patients with PID have access to appropriate care and good quality of life. Since then, IPOPI (the International Patient Organisation for Primary Immunodeficiencies), has been working with national PID patient organisations as well as collaborating with scientific and medical institutions and experts to bring these principles closer to the day-to-day life of individuals with PIDs.

**Method:**

The six PID Principles of Care were revised to consider advances in the field, as well as political developments that had occurred after their initial publication in 2014. Based on this revision the list was updated, and a new principle was added. The six established principles were: diagnosis, treatment, universal health coverage, specialised centres, national patient organisations and registries. Each principle was structured and measured through a series of criteria, and was given the same weight, as they have been considered to all be equally important. Specific weights were attributed to the criteria depending on their relevance and importance to quantify the principle. The index was translated into a survey for data collection: initially involving data from selected countries for a pilot, followed by integration of data from IPOPI’s national member organisations and key countries.

**Results:**

The PID Life Index was developed in 2020 to assess the status of the PID environment and the implementation of the 6 principles worldwide. The Index allows for benchmarking countries either according to a set of principles and criteria or based on the user’s preferences. This can be displayed in an interactive map or through a data visualisation system.

**Conclusion:**

The PID Life Index has been developed successfully and has potential to become an important source of information for PID stakeholders, to increase awareness and information as well as support advocacy initiatives on PIDs nationally, regionally or globally.

## Background

Primary immunodeficiencies (PIDs) are a large and growing group of rare and chronic disorders, caused when some components of the immune system are defective [1]. In 2019, the International Union of Immunological Societies (IUIS) counted 430 single-gene inborn errors of immunity with underlying phenotypes as diverse as infection, malignancy, allergy, autoimmunity and autoinflammation [2]. Given the complexity and wide range of components of the immune system involved for the different types of PIDs, the diagnosis and management of these diseases are complex and require experienced medical specialists in this specific field [1]. This complexity reflects also in the different aspects that need to be taken into consideration when describing comprehensive principles of care for PIDs. It was with this objective in mind that in 2014, a worldwide multi-disciplinary team of specialists, published the PID Principles of Care [3]. The paper incorporated the views of medical experts from all the continents, as well as nurses and patient representatives from IPOPI (the International Patient Organisation for Primary Immunodeficiencies). Together they called for the implementation of these principles, as elements of PID care provision that should be available and implemented in each country. The gold-standard framework for care and management of PIDs included six principles of care that IPOPI promoted amongst its national member organisations to support their implementation.

In 2019, more than 5 years after the original publication in 2014, discussions started regarding how to monitor the implementation of these PID Principles of Care in the different countries, and to track how countries performed in terms of PID care to their patients. IPOPI thus began considering how to assess the level of implementation of these gold-standard principles as well as how to create a tool that could capture the status of the PID healthcare environment in each country. The result of this reflection is the PID Life Index, an interactive tool built on 6 key principles of care for PID. In this development process, the initial 6 principles were revised to take into account advances in the field, resulting in the following 6 principles: PID diagnosis, treatments, universal health coverage, specialised centres, national patient organisations and registries for PIDs.

This paper explains the development of the tool, from the initial inception to the development of the interface as well as the deployment of the data. It is hoped that the tool can be seen as a model for monitoring the healthcare environment of other complex rare diseases.

## Methods; Development of the PID Life Index

### The creation of an index

The PID Life Index was developed in several stages, the first step being to assess the described principles in the 2014 article [3], consider necessary updates and understand how these could be measured through quantitative criteria. For this assessment, IPOPI engaged the expertise of 3 dedicated experts in the field of PIDs, as well as patient representatives, in a working group. These were all members of IPOPI’s Medical Advisory Panel, Board of Directors, or staff, and represented different areas of expertise (e.g., both paediatric and adult PID care) as well as brought experience from different regions. Following the establishment of the working group, the principles and criteria were discussed and assessed during two separate workshops. These discussions led to the agreement on the following topics for the principles: diagnosis, treatment, universal health coverage, specialised centres, national patient organisations and registries:

- **Diagnosis** measures the availability of tests for the diagnosis of PID patients within a given country, as well as the diagnosis rate expressed as a percentage, built based on the theoretical number of PID patients (1:2000) and the known number of patients in that country.
- **Treatment** considers the availability of treatments for patients with PIDs in a given country and the availability of reliable plasma and blood collection infrastructure.
- **Universal health coverage** shows the level of reimbursement or coverage by the national/regional health or social system of a country on diagnostics or treatments for patients with PIDs. This data is provided as a percentage, estimating the part of the diagnostic or treatment that is provided free of charge or reimbursed to patients with PIDs compared to patients’ out of pocket expenses.
- **Specialised centres** for children and adults working within the framework of a national network is considered the best model to address complex rare conditions such as PIDs in their different dimensions (diagnosis, treatment, care provision, transitioning and ageing care). Information is given on the availability of these networks/centres and their specificities for each participating country.
- **National organisations** of patients with PIDs have a significant role to play in healthcare systems. It is well recognised that patient representatives have become experts on their conditions and relevant treatments and bring unique and personal perspectives on the impact of diagnosis and treatment to their communities [3]. Additionally, they work to improve patients’ and healthcare professionals’ awareness and education on these conditions and advocate, so the health authorities adapt the legal framework to these rare conditions.
- **Patient registries** constitute important instruments to serve research in the field of rare diseases and to improve patient care and healthcare planning. They help pool data in order to achieve a sufficient sample size for epidemiological and/or clinical research [4], assess treatments and facilitate/encourage clinical trials.

From the 2014 landmark publication, the only differing principle is universal health coverage, which relates to “managing PID diagnosis and care in all countries” and links the Index with the WHO initiative on Universal Health Coverage [5].

Each of the principles were described by a series of measurable criteria (Table 1). Each principle has been given the same weight, as they have been considered to all be equally important. Specific weights were attributed to the criteria depending on their relevance and importance to quantify the principle. This allows for observing progress in the implementation, with the final objective of seeing countries scoring steadily higher in every principle over time, indicating that the PID environment in the country is improving. The Index allows for updates and future calibration in line with the global evolution of the PID environment.

**Table 1:** Principles and criteria used to build the PID Life Index

Principle	Criteria
PID diagnosis	Diagnosis rate
	Biological diagnosis availability
	Genetic diagnosis availability
	Prenatal diagnosis availability
	Newborn screening for SCID
Treatments	Anti-infectious availability
	Immunoglobulin availability
	Vaccine availability
	Curative treatments
	Biological and targeted therapies
	Plasma collection
Universal health coverage	Diagnosis reimbursement
	Anti-infectious reimbursement
	Immunoglobulin reimbursement
	Vaccine reimbursement
	Curative treatments reimbursement
	Biological and targeted therapies reimbursement
Specialised centres	National PID specialised centre/network
	Adult PID services
	Transition care
National patient organisations	Established national group in the country
	Professional paid staff
	Main working areas
Registries	National PID registry
	Bone marrow donor registry

### Creation of a PID Life Index database

Once the principles and criteria were defined, they were translated into a questionnaire used to gather data from the different countries. This questionnaire was tested in a pilot phase in a reduced number of countries with different development levels, based in different regions in the world, to perfect and validate it. Once the pilot questionnaire had been sent out to a country respondent, an interview was scheduled to document the respondent’s reaction to the questionnaire, as well as to respond to potential questions that could arise. This approach was chosen to ensure an open dialogue and to understand if there were any confusing concepts or questions included in the questionnaire. Given the fact that the PID community has a common medical language, the doubts from the respondents were limited and mainly concerned the principle on Universal Health Coverage, as national health care systems, including the system for reimbursement, are not structured in the same way in every country. To respond to this, and to allow for more flexibility on this principle, an option for the respondents to include a nation-specific comment was included in the questionnaire.

Following this, the questionnaire was amended accordingly, allowing for the working group to produce a validated questionnaire applicable worldwide, with limitations. The data collection process was then enlarged to include all IPOPI national patient organisations and those countries in which IPOPI has specialised medical relationships. The information received was provided by IPOPI’s counterparts to the best of their knowledge. This questionnaire will also be used for a yearly revision of the data; however, data are open for revision at any time.

### Display of the data

**Fig. 1:**
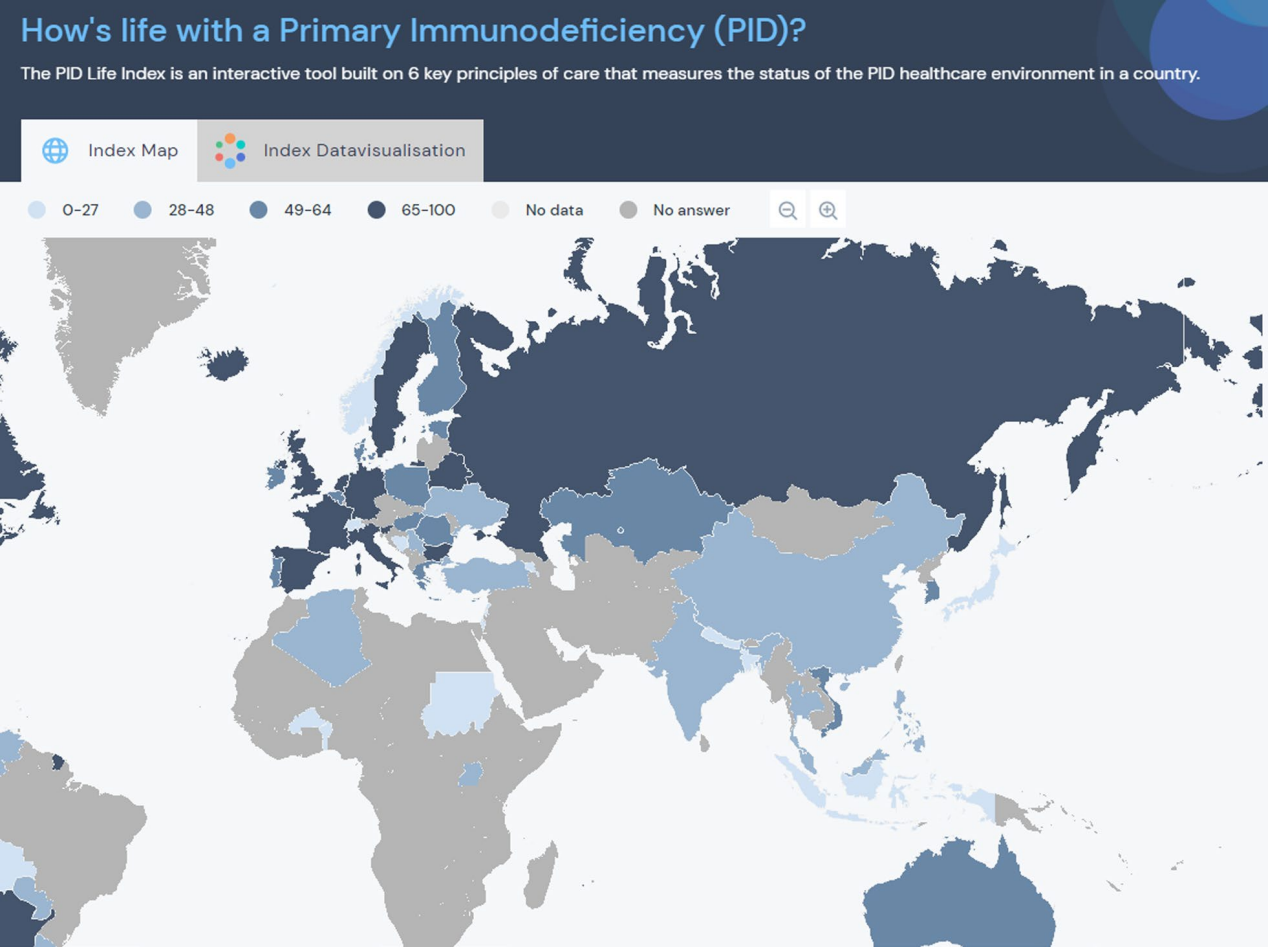
Overview of the global index

**Fig. 2:**
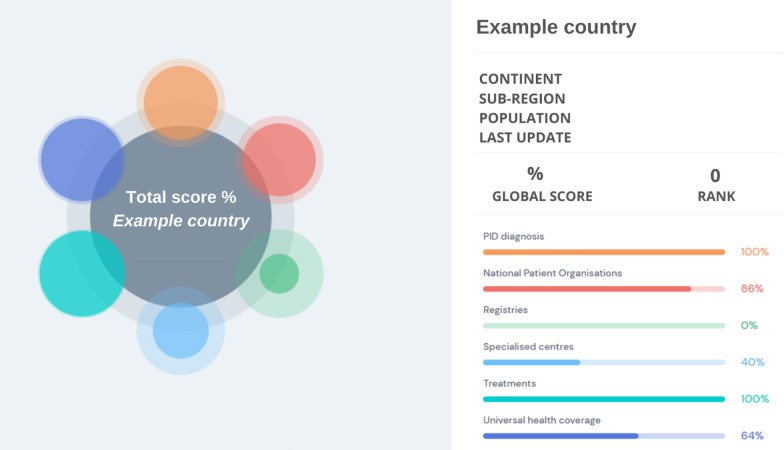
Example of a country page

The database with the collected information was uploaded to an online platform that displays information on the PID care globally through different settings; a web-based visualisation system and a world map presenting the global index (Fig. 1); the different principles as well as the criteria; and by country. In addition, each country has a devoted country page (Fig. 2) where users can learn more closely about the national PID environment in a given country. The PID Life Index supports web-based resource mapping of aggregated (global, by continent) and individual data, including for example showing relations between countries within the same region for each principle.

Additional features of the tool include: (1) a list of specialised centres for PIDs for children and adults and their location within each country, (2) a list of immunoglobulin replacement therapies registered per country, (3) a country page that summarises the data that are available in the database and allows for the provision of further comments detailing the specificities of the country if need be, (4) a glossary to help centralise the information on the terms used in a simple manner for the reader.

The PID Life Index is freely accessible on IPOPI’s website or through this link [6] on a computer, tablet or mobile phone.

## Results

The PID Life Index offers a comprehensive and holistic overview of the PID environment in different countries and regions of the world. The data collection questionnaire was tested in a pilot phase with a limited number of countries and amended according to the feedback given. Following its validation, the questionnaire was broadly disseminated, and data was collected from several of IPOPI’s national member organisations, as well as from countries where IPOPI had known PID expert contacts. Following this collection phase, the data were fed into the PID Life Index database, allowing for the creation of the PID Life Index.

**Fig. 3:**
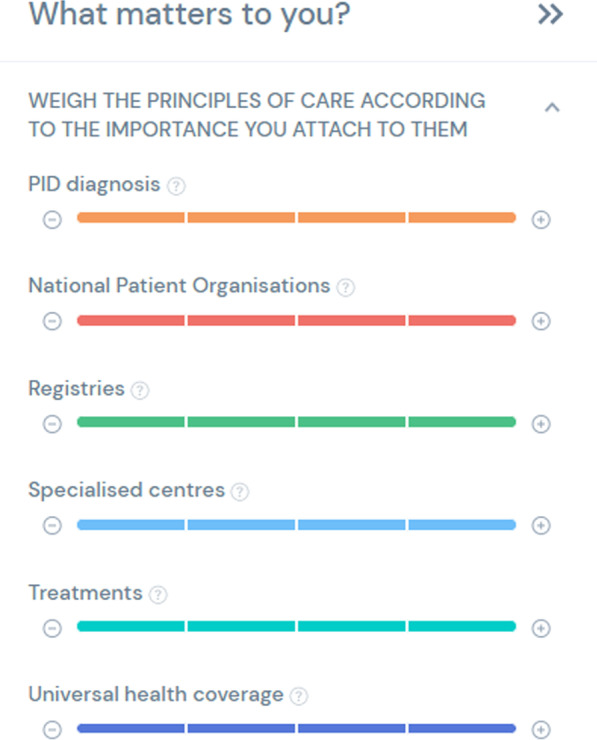
Principle ranking feature

The overall result is a web-based tool that allows for (1) handling large amounts of data on key indicators for PID patients, (2) describing and comparing life with PID within countries and regions easily either according to all the principles or any principle(s) and/or criteria that matter most to the user. The data is displayed through a map or through a data visualisation system that ranks countries according to their score in the Index. This visualisation system provides three different options to see the countries ranked: either by displaying countries in a ranking from high to low, in a list from highest scores to the lowest or by alphabetical order. The images selected for the data visualisation system allow for the inclusion of the overall score of a given country and the display of the score of the 6 individual principles. The user can then decide to have the ranking of the countries according to the importance they want to give to each principle (Fig. 3). The score of all the selected countries is immediately calculated according to this preference and the selection of principles chosen by the user.

The Index world map is the page shown by default when the user enters the interface (Fig. 1). Initially, the map displays the Index score of the countries calculated on the basis of all 6 defined principles, each of them having the same weighting. The user can then decide which specific principle is of most interest and, within the principle, certain criteria. The user interface is currently available in two languages (English and Spanish).

The PID Life Index constitutes the first known attempt to gather and compile information on the PID environment according to the 6 principles of care for PIDs, for any organisation in the field of PIDs or rare diseases in general. The overall objective for the Index is three-fold: (1) to support patients, patient advocates and healthcare professionals in their advocacy efforts to improve the PID care in a given country or region, (2) to help understand the current situation of patients with PIDs and their healthcare professionals or scientists in a specific country, (3) to encourage fruitful cooperation between all the stakeholders who each in their field contribute to the best possible situation for patients with PID in a country: patient organisations, health care professionals, regulators and health authorities, industry, experts in health economics.

## Discussion

Often in the field of primary immunodeficiencies and of rare diseases in general, patient organisations, medical experts, and stakeholders in the field face the lack of comprehensive data, rendering it impossible to understand the situation in a given country or group of countries according to the same criteria. Moreover, web-based information technology has revolutionised many areas of our daily lives, but its application to the health sector is still limited [7, 8].

The PID Life Index constitutes the first internet-based technology initiative that aims at displaying data on a set of comprehensive principles describing the level of care for patients with PIDs in the different countries and regions of the world. The provision of a set of defined and detailed principles facilitates the comparison across different countries and has the potential to be used by national PID patient organisations or healthcare professionals in their advocacy and awareness-raising activities at the national level as a way of benchmarking their country in comparison to other countries in the region or more widely. This is one of the most relevant uses of the tool: to provide a global data overview to patient representatives and healthcare experts for their discussions with policy makers, payers or any relevant decision-maker in their countries to improve the quality of life and outcomes of patients with PIDs and the healthcare infrastructures, allowing for a better provision of care for PID patients.

The aim of the PID Life Index is not only to inform PID stakeholders and allow countries to align on best practices, but also to support national advocacy efforts aimed at improving diagnosis and care for these patients. So, although PIDs are heterogenous in nature and diagnosis and management are complex, addressing them as one family of diseases will allow for stronger advocacy initiatives. However, the heterogenous nature of PIDs has been considered when establishing the criteria for each principle, for example by providing the user with access to information on specific therapies and allowing for targeted advocacy efforts for specific PIDs.

The PID Life index approach is not only to compile and use the resource data from IPOPI’s collaborators and to facilitate networking, but also to work alongside national expert PID medical centers and registries to improve local data quality and coverage, and analytical capacities of the tool. It is hoped to reinforce the partnership between patients’ associations and health care professionals. This is a first-of-its-kind care status measurement index in the rare diseases field, with potential to become a model for aggregating and presenting data for other complex rare diseases. It is of extra importance when considering rare diseases, where lack of data (or shattered data) remains a great obstacle and where stakeholders could truly benefit from an index of this kind.

The PID Life Index is also the first attempt to provide a global harmonized educational approach to what are the gold-standard principles of care for PIDs, and what patients with PIDs and medical experts should focus on to improve the environment in their country. This tool also establishes a global language that attempts to bridge the cultural and societal differences across countries. It is anticipated that the tool will improve over time, not only with refinements of the tool itself, but also from the successive learning of the national patient organisations who will improve their knowledge and expertise regarding the different principles and thus also their ability to report on them.

The data gathered in the PID Life Index will be regularly updated to continue reflecting the national realities of patients with PIDs. IPOPI has envisaged calling for a yearly update of the instrument to ensure that the information included in the PID Life Index is accurate and up to date. In addition, there are plans to extend the geographical scope by including new countries. With future rounds of revision, we anticipate that the Index will need to be updated to reflect the state-of-the-art of the PID environment and science. It is also foreseen to open the country pages to the national patient organisation representatives to access so that they can include further information than was included in the initial survey.

The amount of data gathered for the launch of the Index was dependent on the participation of the countries invited to contribute. Data were provided for those countries in which IPOPI has a national PID patient organisation or where there are good and well-respected medical experts in the field of PIDs. Participation in the project is voluntary and some countries decided not to take part or were not ready at the time to provide the answers required. It is expected that, in the envisaged annual reviews of the data, new countries will contribute to the Index and allow for an even better understanding of the PID environment.

### Interpretation and alternative perspectives

One of the identified potential limitations of the PID Life Index relates to the personal and cultural perspective of the person/persons answering the survey. The chosen approach to collect data on the different countries was to utilise IPOPI’s network of national member organisations or go through a medical contact in countries where no patient organisation existed. The answers provided were given to the best of the respondent’s knowledge and in good faith. Depending on how the respondent approached the questionnaire, they could consider the tool as a way of promoting the country. This has been partially addressed when the respondents have seen the data of their countries in comparison to the reality of the patient with PID in the country. This system has its caveats, as it relies on the perspective of one individual, especially in countries where only one medical expert was known. Cultural differences and interpretations when replying to the questionnaire have also played a part in the responses received and the understanding of some of the questions has been different depending on the regions of the world the respondents were based. As a response to this point, the Index offers the possibility for users to easily contact IPOPI with questions and comments regarding the data. Depending on the feedback, the country contact is then consulted, and the specific data is reviewed and updated should it be deemed necessary.

Another potential shortcoming of the instrument is that the national perspective is not always able to reflect the reality in certain countries. The PID Life Index aims at providing a general perspective in a given country. It currently does not allow for providing information on specific regions/provinces that may perform better than others if the territorial system in the country is decentralised. It also does not consider the potential discrepancies between major cities in a country and the rest of the country. The fact that respondents to the survey are asked about the national situation may, therefore, be biased depending on where the respondent is based within the country. This may be solved when the country page is opened for the direct input of the national patient organisation, so that additional details and information not yet covered can be added and a wider perspective than the one included in the questions can be added.

One of the elements that was raised during the development of the questionnaire and again when validated by the provision of answers was the existing discrepancies between national law or policy and the day-to-day life of patients with PIDs. This caveat has been pointed out repeatedly by our contacts in the different countries: what the health and/or social provisions in a given country say may greatly differ from the reality of patients with PIDs and their families. This is especially obvious when asked about coverage or reimbursement of diagnosis and/or treatment for patients with PIDs. In many cases, national laws provide for the coverage of diagnosis and/or treatment, although these provisions are not enforced or not respected for patients with PIDs. This is also shown by the activities developed by the national patient organisations, where many have indicated that they provide legal support to their members. This could be highlighted in the future when the different patient organisations can directly enter information about their country or their activities.

## Conclusion

The PID Life Index is a very ambitious project that aims at providing a vision of the reality of patients living with PIDs in the world, depending on the country or region they live in. Based on a reflection process arising from the PID Principles of Care, the Index allows for their translation into concrete indicators that reflect the lives of patients with PIDs from a patient perspective. The information provided is measurable across countries and regions, allowing for comparisons that will help inform patients, patient representatives, medical experts, and other users where they stand, help them make informed choices and support their advocacy efforts at national level. The implementation of the PID Life Index will also make visible potential gaps relevant for the improvement of national and international policies on PID care, as well as to better assess the compatibility of the care principles across countries. Despite its limitations, this initiative will certainly help to identify ways to improve information processes on PID care through internet-based technology.

## Data Availability

The datasets gathered for this project are available in the PID Life Index [https://pidlifeindex.ipopi.org/].
